# To the Editors of the Medical and Physical Journal

**Published:** 1800-06

**Authors:** Andrew Blackburn

**Affiliations:** Hereford


### *To the Editors of the Medical and Phyſical Journal.*

GENTLEMEN,

BEING fully ſenſible of the diſguſting appearance produced in mixtures by an unſucceſsful combination of ſpermaceti with water, I was much pleaſed to read in your 13th number, (page 263) an attempt to meliorate it. But though the contrivance is ingenious, I am far from being of opinion that the method recommended will be adopted in practice, on account of the ſtrong rancid ſmell, and diſagreeable taſte, produced in the mixture by means of the heat uſed in the proceſs; a circumſtance of no ſmall importance to a certain claſs of patients, whoſe capricious palates it is oftentimes exceedingly troubleſome and difficult to pleaſe. I am further of opinion that the heat employed, (together with the additional proportion of yolk of egg, which will be found indiſpenſably neceſſary to fit the ſpermaceti for the reception of the water) would diſpoſe the mixture to become ſour too readily, thereby not only rendering it diſagreeable to the taſte, but injurious to the ſtomach. Reſpecting the ſmoothneſs and uniformity of the mixture, I think if the following formula be uſed, it will be found to ſucceed in every reſpect as well, and as ſoon as the one in queſtion.

Spermaceti and double refined ſugar, each two drams; rectified ſpirits of wine, thirty drops; yolk of egg, a very ſmall quantity; diſtilled or common water, half a pint.

The ſpermaceti and rectified ſpirits are firſt rubbed together a ſhort time, the ſugar is then added, and the trituration continued ſome time longer; then the egg, and laſtly the water.

The above recipe, in the hands of a ſkilful operator, is almoſt invariably found to anſwer the purpoſe of forming an uniform mixture, unaccompained by any of the diſagreeable conſequences above alluded to, and will, with the addition of any ſpirituous water, keep a ſufficient length of time. I am,

GENTLEMEN,

Your very humble ſervant,

*Hereford, March* 10, 1800. ANDREW BLACKBURN.

